# Common Chemical Inductors of Replication Stress: Focus on Cell-Based Studies

**DOI:** 10.3390/biom7010019

**Published:** 2017-02-21

**Authors:** Eva Vesela, Katarina Chroma, Zsofia Turi, Martin Mistrik

**Affiliations:** 1 Institute of Molecular and Translational Medicine, Faculty of Medicine and Dentistry, Palacky University, Hnevotinska 5, Olomouc 779 00, Czech Republic; eva.vesela@upol.cz (E.V.); katarina.chroma@upol.cz (K.C.); zsofia.turi01@upol.cz (Z.T.); 2 MRC Laboratory for Molecular Cell Biology, University College London, London WC1E 6BT, UK

**Keywords:** replication stress, cisplatin, aphidicolin, hydroxyurea, camptothecin, etoposide, cancer

## Abstract

DNA replication is a highly demanding process regarding the energy and material supply and must be precisely regulated, involving multiple cellular feedbacks. The slowing down or stalling of DNA synthesis and/or replication forks is referred to as replication stress (RS). Owing to the complexity and requirements of replication, a plethora of factors may interfere and challenge the genome stability, cell survival or affect the whole organism. This review outlines chemical compounds that are known inducers of RS and commonly used in laboratory research. These compounds act on replication by direct interaction with DNA causing DNA crosslinks and bulky lesions (cisplatin), chemical interference with the metabolism of deoxyribonucleotide triphosphates (hydroxyurea), direct inhibition of the activity of replicative DNA polymerases (aphidicolin) and interference with enzymes dealing with topological DNA stress (camptothecin, etoposide). As a variety of mechanisms can induce RS, the responses of mammalian cells also vary. Here, we review the activity and mechanism of action of these compounds based on recent knowledge, accompanied by examples of induced phenotypes, cellular readouts and commonly used doses.

## 1. Introduction

The DNA molecule always has to keep the middle ground: it must be sufficiently rigid to maintain correct genetic information while at the same time available for ongoing processes. DNA is particularly vulnerable to insults during replication, a process where a copy of the genome is generated [1]. Replication must be tightly regulated because it is essential for genome integrity, and therefore the fate of a new cellular generation. Accurate coordination of several cellular pathways is needed to provide sufficient energy and material supply, precise timing and functional repair to overcome arising difficulties [1].

Transient slowing or disruption of replication fork (RF) progression is called replication stress (RS), which can be caused by a limitation of important factors and/or obstacles caused by intrinsic and extrinsic sources [2]. Intrinsic sources of RS involve the physiological properties of the DNA molecule, such as regions of heterochromatin structure, origin-poor regions or sites rich in some types of repetitive sequences [3,4,5]. Other intrinsic sources of RS are generated by deregulated pathways that cause over- and under-replication [6,7,8], re-replication (also known as re-duplication) [9,10], or by transcription and replication machinery collisions [9].

**Figure 1:**
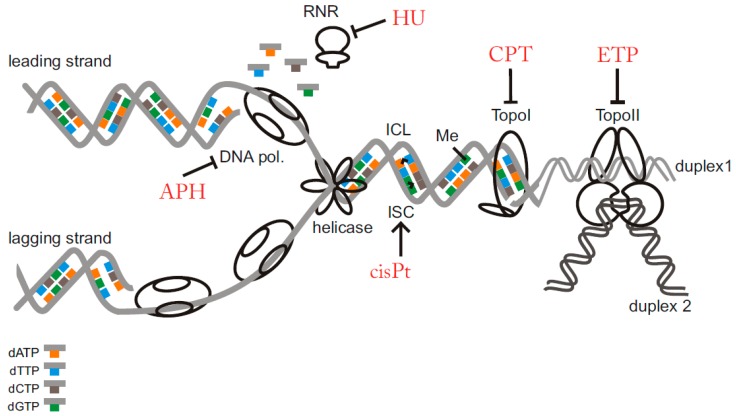
Schematic view of the most common lesions causing replication stress. In the scheme, several important replication stress (RS) inducing factors are illustrated: intra-strand crosslink (ISC), inter-strand crosslink (ICL), alkylated/modified base (Me) and inhibition of replication related enzymes. Compounds further described in the review are marked by red colour. RNR: ribonucleotide reductase; DNA pol.: DNA polymerase; TopoI: topoisomerase I; TopoII: topoisomerase II; APH: aphidicolin; HU: hydroxyurea; CPT: camptothecin; ETP: etoposide; cisPt: cisplatin; dATP: deoxyadenosine triphosphate; dTTP: deoxythymidine triphosphate; dCTP: deoxycytidine triphospahte; dGTP: deoxyguanine triphosphate.

The most common extrinsic sources of RS are all wavelengths of ultraviolet radiation (UV) [11], ionising radiation (IR) [12] and special genotoxic chemical compounds [13] which are the main focus of this review. RS-inducing chemicals can cause a broad spectrum of DNA lesions. Alkylating agents such as methyl-methane sulfonate (MMS) [14], temozolomide and dacarbazine [15] directly modify DNA by attaching an alkyl group that presents an obstacle to RF progression. Moreover, the bifunctional alkylating compounds (e.g., mustard gas) can cause the crosslinking of guanine nucleobases [16,17] that violate the DNA structure even further [18]. Typical crosslinking agents introduce covalent bonds between nucleotides located on the same strand (intrastrand crosslinks), like cisplatin, or opposite strands (interstrand crosslink), like mitomycin C, and psoralens [18]. Crosslinks make the strands unable to uncoil and/or separate and physically block RF progression [19]. Even a small amount of unrepaired crosslinks (approx. 100–500) is reported to be lethal to a mammalian cell [20]. Furthermore, single-strand DNA breaks (SSB) and double-strand DNA breaks (DSB) represent a specific problem for ongoing replication which is well manifested by increased sensitivity of replicating cells towards radiomimetic compounds (e.g., neocarzinostatin) [21]. Other compounds do not damage the DNA structure directly but rather interfere with replication-related enzymes. Aphidicolin, an inhibitor of replicative DNA polymerases leads to uncoupling of the replicon and generation of long stretches of single-stranded DNA (ssDNA) [22]. After hydroxyurea treatment, an inhibitor of ribonucleotide reductase (RNR), the metabolism of deoxyribonucleotide triphosphates (dNTPs) is disturbed, and subsequently, the RF progression is blocked [23]. Camptothecin and etoposide, inhibitors of topoisomerase I and topoisomerase II respectively, prevent DNA unwinding and halt relaxation of torsional stress [24,25]. The most common sources of RS are illustrated in Figure 1.

Several repair pathways are essential for rapid elimination of DNA distortions and lesions introduced by the action of RS inducing compounds [26]. Removal and replacement of single base damage (e.g., oxidised and alkylated bases), is performed by base excision repair (BER) [27]. More extensive damage affecting several adjacent bases is repaired by nucleotide excision repair pathway (NER). NER is essential for repair of UV-induced damage such as cyclobutane pyrimidine dimers, or pyrimidine-pyrimidone (6-4) photoproducts and also needed for crosslinks removal caused by for example cisplatin [28]. Single-strand break repair in higher eukaryotes rely on poly(ADP-Ribose) polymerase 1 (PARP1) and X-ray repair cross complementing 1 (XRCC1) depedent recognition of the lesion, followed by end processing and ligation [29]. Double-strand breaks (DSBs) are processed by either homologous recombination (HR), or non-homologous end-joining (NHEJ). HR is active predominantly in S and G2 phases using the sister chromatid as a template for repair with high fidelity [30]. NHEJ, considered as an error prone pathway, performs DSB repair in all cell cycle stages more rapidly by direct ligation of two unprocessed (or minimally processed) DNA ends [31].

All previously described specific structures and concomitant DNA lesions can challenge the progression of RF. If the RF encounters a lesion which the replicative polymerase is unable to process as a template, it becomes stalled [32]. Stalled RFs are vulnerable structures and may undergo spontaneous collapse which leads to DSBs and genomic instability (GI) [33,34]. To avoid the harmful consequences of stalled forks, several mechanisms—DNA damage tolerance pathways (DDT)—exist to bypass the lesions and enable fork restart. One well-described process of DDT is translesion synthesis (TLS). TLS promotes “polymerase switch” from the replicative polymerase to translesion polymerases, which are able to continue replication across the lesion. TLS polymerases possess low processivity and fidelity towards the template DNA strand. Therefore TLS is often referred to as the error-prone pathway of DDT [32,34,35,36]. Among the DNA lesions which block the progression of RFs, interstrand crosslinks (ICLs) belong to the most challenging to bypass [37]. Thus, a whole group of proteins called Fanconi anaemia (FA) proteins evolved to govern the bypass and the repair of ICLs. The FA network promotes the unhooking of the ICL by specific endonucleases, bypassing the lesion by TLS polymerases or the repair by HR [5,6,7]. Patients with a defect in the FA protein family suffer from premature ageing, show increased sensitivity to DNA crosslinking agents (e.g., cisplatin, mitomycin C) and predisposition to certain types of cancers due to increased GI [38,39,40]. Although the FA pathway is involved mainly in ICL repair, it contributes more generally to initial detection of RF arrest, processing and stabilisation of the forks and regulation of TLS [41,42].

DNA damage bypass can occur in an error-free manner through the activation of the other branch of DDT, called template switching (TS). The process utilises the newly synthesised strand of the sister duplex, using it as an undamaged template. TS can be promoted either by fork regression or by strand invasion mediated by HR [34,36,43,44]. RF restart can also be achieved by firing nearby dormant replication origins or by repriming events leaving behind lesion containing single-stranded DNA (ssDNA) gaps which are subsequentially processed by DTT pathways [45,46,47,48,49,50]. Altogether, these processes ensure the rapid resumption of DNA synthesis, preventing prolonged fork stalling and the potentially deleterious effects of replication fork collapse. However, upon persisting RS, or non-functional RS response, the RF may fail to restart and collapse, most probably due to destabilised, dysfunctional or displaced components of replication machinery [1,50,51,52,53,54]. Prolonged stalled replication forks are targeted by endonucleases followed by recombination-based restart pathways [55,56].

Among the features of RS belong accumulation of long stretches of ssDNA [46,57], resulting from the uncoupled activity of DNA polymerase and progression of DNA helicase [58,59]. The persisting ssDNA is rapidly coated by replication protein A (RPA) that in turn generates the signal triggering the checkpoint response through activation of Ataxia telangiectasia Rad3-related (ATR) checkpoint kinase [60,61,62,63]. Once activated, ATR and its downstream target checkpoint kinase 1 (CHK1) help the cell to faithfully complete DNA replication upon RS [52,53,64]. In addition, ATR as the central RS response kinase contributes to the stabilisation and restart of the stalled forks even after the stress has been removed [65]. The ATR-CHK1 pathway is responsible for cell cycle inhibition, suppression of new origin firing, DNA repair and to the overall improvement of cell survival [62,66]. The role of Ataxia telangiectasia mutated (ATM), another important checkpoint kinase, upon RS conditions is not as clear and straightforward as of ATR. ATM is preferentially activated by DSBs which are generated in later stages after RS induction, mostly after the RF collapse [67,68]. There is suggested interplay between ATM and ATR during replication stress which becomes apparent under concomitant depletion of both kinases [68]. Interplay between ATM, Werner helicase (WRN) and Bloom helicase (BLM) is needed for the resolution of replication intermediates and HR repair pathway that is important for RF restart [69,70].

Chronic replication stress conditions, particularly in the absence of proper DNA repair pathway and/or non-functional checkpoint responses might result in the transfer of RS-related DNA alterations to daughter cells, inducing mutations, GI and fuelling tumourigenesis [1].

From this point of view, the RS is a strong pro-carcinogenic factor driving selective pressure for acquisition of mutations overcoming cell cycle arrest or apoptosis [71,72]. This further leads to the progression of malignant transformation and faster selection of mutations allowing development of resistance to cancer treatment [73].

However, cells typically react on the prolonged exposure to RS by triggering mechanisms leading to permanent cell cycle arrest known as cellular senescence or apoptosis [74,75] acting as a natural barrier against tumour progression [76].

Several hereditary syndromes are linked to enhanced RS and GI. The spectrum of exhibited symptoms is broad and includes premature ageing, growth retardation, neurodegeneration, immunodeficiency, cancer predisposition and others. The disorders like Seckel syndrome (deficiency in ATR kinase) [77], Ataxia telangiectasia caused (loss of ATM kinase) [78], Xeroderma pigmentosum (XP); various defects in XP protein family group) [79] are caused by aberrations in DNA damage recognition and repair enzymes [80]. Bloom and Werner syndrome (deficiency of BLM and WRN helicase, respectively) [81,82], Fanconi anaemia (FA; mutations in FA pathway proteins) [83,84], or Rothmund-Thomson syndrome (defects in RECQ like helicase 1 protein) [85,86] are related to failure of replication fork progression and restart.

In general, RS is a potent inducer of variety of hereditary and non-hereditary diseases, including the oncogenic transformation. The knowledge and understanding of the processes during RS are crucial for choosing the most efficient therapy. The in vitro-based cell studies involving models of chemical induction of RS are unique source of information about molecular interactions and undergoing mechanisms. For this review five compounds were chosen, all of them are commonly used for cell-based experiments to induce RS. Several aspects are discussed in detail: mechanism of action aimed at replication interference, proper dosing and common experimental setups. A brief overview of the medical use and important practical hints for laboratory use are also included.

## 2. Compounds

### 2.1. Cisplatin

**Figure 2:**
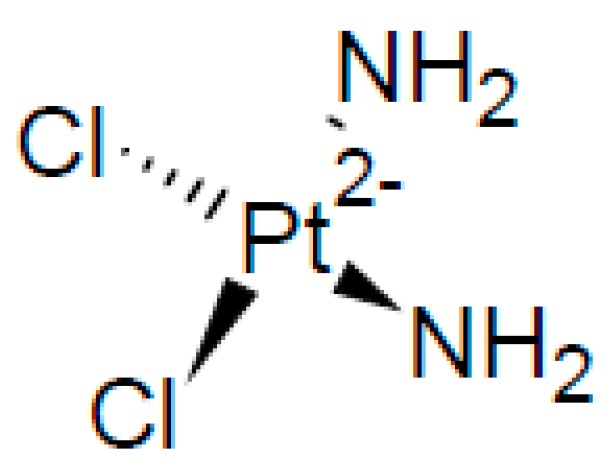
Cisplatin structure.

Cisplatin (cisPt) is an inorganic platinum complex first synthesised by Italian chemist Michel Peyrone and originally known as ‘Peyrone’s chloride’ (Figure 2). The cytostatic activity of cisPt was first reported by Barnett Rosenberg and co-workers in 1965 following accidental discovery of *Escherichia coli* growth inhibition induced by the production of cisPt from platinum electrodes [87]. It is generally considered as a cytotoxic drug for treating cancer cells by damaging DNA and inhibiting DNA synthesis. cisPt is a neutral planar coordination complex of divalent platinum [88] with two labile chloride groups and two relatively inert amine ligands. The *cis* configuration is necessary for the antitumour activity [89], 3D structure of monofunctional cisPt bound to DNA structure can be found here [90].

#### 2.1.1. Mechanism of DNA Damage Induction

The cytotoxicity of cisPt is known to be due to the formation of DNA adducts, including intrastrand (96%) and interstrand (1%) DNA crosslinks, DNA monoadduct (2%) and DNA–protein crosslinks (<1%) [91]. These structural DNA modifications block uncoiling and separation of DNA double-helix strands, events both necessary for DNA replication and transcription [92]. Inside a cell, cisPt forms an activated platinum complex, which triggers a nucleophilic substitution reaction via an attack on nucleophilic centres on purine bases of DNA, in particular, *N*7 positions of guanosine (65%) and adenosine residues (25%) [93]. The two reactive sites of cisPt enable the formation of the most critical crosslink between two adjacent guanines (1,2-d(GpG)), resulting in the formation of DNA intrastrand crosslinks [94]. Also, platinum can align to guanine bases on the opposite DNA strand, thus creating DNA interstrand crosslinks, present in lower percentage [95]. These cisPt crosslinks create severe local DNA lesions that are sensed by cellular proteins, inducing repair, replication bypass or triggering apoptosis [96]. Several protein families can recognise cisPt–DNA adducts, including nucleotide excision repair (NER) proteins [97], homology-directed repair proteins (HDR) [98], mismatch repair (MMR) proteins [99] and non-histone chromosomal high mobility group proteins 1 and 2 (HMG1 and HMG2) [100]. The intrastrand cisPt structural alteration stalls RNA polymerase II. It is recognised and efficiently repaired by global genome NER (GG-NER) or its transcription-coupled sub-pathway (TC-NER) [101]. The second DNA repair system predominantly involved in coping with cisPt–DNA adducts is error-free HDR, which removes DNA DSBs remaining after cisPt adduct removal [98]. In contrast to the previously mentioned repair pathways that increase cell viability, MMR proteins have been shown to be essential for cisPt-mediated cytotoxicity [99]. cisPt is reported to enhance interactions between MMR proteins MLH1/PMS2 (MutL homolog 1/PMS1 homolog 2, MMR component) and p73, triggering apoptosis [102]. Therefore, mutations in MMR genes are known to be associated with cisPt resistance [103]. HMG1 and HMG2 recognise intrastrand DNA adducts between adjacent guanines, affecting cell cycle events and subsequently inducing apoptosis [100].

In addition to the previously mentioned repair proteins, specialised translesion DNA polymerase eta (η) can be loaded onto sites of cisPt–DNA adducts promoting TLS repair pathway [104]. cisPt also induces dose-dependent reactive oxygen species (ROS), which are responsible for the severe side effects of platinum-based therapy, including nephrotoxicity and hepatotoxicity [105]. When overwhelming the reduction capacity of the cell, cisPt-induced ROS might lead to lipid peroxidation, oxidative DNA damage, altered signal transduction pathway and calcium homoeostasis failure [105]. Extensive unrepaired cisPt-induced DNA damage can proceed to apoptotic cell death mediated by various signal transduction pathways, including calcium signalling [106], death receptor signalling [107] and activation of mitochondrial pathways [108]. At least two main pathways have been proposed to mediate cisPt-induced apoptosis in vitro. One involves the critical tumour suppressor protein p53 directly binding to cisPt-modified DNA [109] and promoting apoptosis via several mechanisms. p53 binds and counteracts the anti-apoptotic B-cell lymphoma-extra large (Bcl-xL) [110], contributes to inactivation of nutrient sensor AMP-kinase (AMPK) [111], activates caspase-6 and -7 [112] and the pro-apoptotic Bcl-2 family member PUMAα in renal tubular cells [113]. However, the role of p53 in response to cisPt seems to be controversial, as it has been described to contribute to cisPt cytotoxicity [114] and also to be involved in cisPt resistance in different cancer models [115]. The other cisPt-induced apoptotic pathway is mediated via a pro-apoptotic member of the p53 family, p73. cisPt has been shown to induce p73 in several cancer cell lines [116], which cooperates with the MMR system and c-Abl tyrosine kinase, known to be involved in DNA damage-induced apoptosis [117]. In response to cisPt, c-Abl phosphorylates p73, making it stable [118], and increases its pro-apoptotic function by binding transcription coactivator p300, which triggers transcription of pro-apoptotic genes [119]. Moreover, p73 forms a complex with c-Jun N-terminal kinase/stress-activated protein kinase (JNK), leading to cisPt-induced apoptosis [120]. Intrinsic signaling pathways involved in cisPt driven apoptosis include Akt [121], protein kinase C [122,123], and mitogen activated protein kinases—MAPK (e.g., extracellular signal-regulated kinases; ERK) [124,125,126], JNK [127,128,129] and p38 [130].

#### 2.1.2. Other Effects

Besides DNA, the primary target of cisPt in cells, there is some evidence for the involvement of non-DNA targets in cisPt cytotoxicity [131]. cisPt interacts with phospholipids and phosphatidylserine in membranes [132], disrupts the cytoskeleton and alters the polymerization of actin, probably due to conformational changes resulting from the formation of Pt–S bonds [133]. MicroRNAs (miR), which play a role in posttranscriptional gene silencing, have been shown to be involved in the modulation of cisPt resistance-related pathways in different cancer models. miR-378 was shown to reverse resistance to cisPt in lung adenocarcinoma cells [134], whereas miR-27a was shown to be upregulated in a multidrug resistant ovarian cancer cell line, contributing to cisPt resistance [135]. miR-21 increases the cisPt sensitivity of osteosarcoma-derived cells [136]. For references to particular studies using cisPt, refer to Table 1.

#### 2.1.3. Solubility

cisPt (molecular weight (MW) 300.05 g/mol) is water soluble at 2530 mg/L (at 25 °C), saline solution with a high chloride concentration (approx. 154 mmol/L) is recommended. In the absence of chloride, the cisPt chloride leaving group becomes aquated, replacing the chloride ligand with water and generating a mixture of species with increased reactivity and altered cytotoxicity [150,151]. Commonly used solutions for laboratory use are aqueous-based solutions in 0.9% NaCl (0.5 mg/mL), pH 3.5–5. Dissolved cisPt may degrade over a short time, the storage of aliquots is not recommended. However, the stability at −20 °C in the dark is reported to be 14 days. Solutions (in 2 mM phosphate buffered saline buffer with chloride concentration 140 mmol/L) stored at 4 °C should be stable for 7–14 days [152]. Undiluted cisPt is stable in the dark at 2–8 °C for several months [121,153]. Dimethyl sulfoxide (DMSO) can also be used for cisPt dilution, however it is not recommended. The nucleophilic sulphur can displace cisPt ligands, affecting the stability and reducing cisPt cytotoxicity [154]. DMSO introduced in combination studies with cisPt does not affect its activity [152].

#### 2.1.4. Medical Use

Following the start of clinical trials in 1971, cisPt, marketed as Platinol (Bristol-Myers Squibb, New York, USA), was approved for use in ovarian and testicular cancer by the Food and Drug Administration (FDA) in 1979 [155]. cisPt is considered one of the most commonly used chemotherapy drugs for treating a wide range of malignancies, including head and neck, bladder, oesophagal, gastric and small cell lung cancer [156,157]. Moreover, cisPt has been shown to treat Hodgkin’s [158] and non-Hodgkin’s lymphomas [159], neuroblastoma [160], sarcomas [161], multiple myelomas [162], melanoma [163], and mesothelioma [164]. cisPt can reach concentrations of up to 10 μg/mL in human plasma [165]. cisPt is administrated either as a single agent or, in the main cases, in combination with other cytostatics (e.g., bleomycin, vinblastine, cyclophosphamide) or radiotherapy for the treatment of a variety of tumours, e.g., cervical carcinoma [153]. The most important reported side effect is nephrotoxicity, due to preferential accumulation and persistence of cisPt in the kidney [166], later ototoxicity and bone marrow depression. Pharmacokinetic and pharmacodynamic studies have shown that a maximal steady state cisPt plasma concentration of between 1.5 and 2 μg/mL has the most effective chemotherapeutical effect with minimal adverse nephrotoxicity [167]. Many cancers initially responding to cisPt treatment could become later resistant. Mechanisms involved in the development of cisPt resistance include changes in cellular uptake, drug efflux, drug inactivation by increased levels of cellular thiols, processing of cisPt-induced damage by increased NER and decreased MMR activity and inhibition of apoptosis [99,168]. To boost platinum drug cytotoxicity, overcome its resistance and achieve a synergistic effect, new platinum-based drugs, as well as their combinatorial therapy with other antineoplastic agents were developed for cancer treatment [169]. Besides of cisPt derivatives as carboplatin and oxaliplatin, are currently being used in the clinical practice, while nedaplatin, lobaplatin and nedaplatin acquired limited approval in clinical use [170,171]. Recent discoveries described the combination of cisPt with PARP inhibitor olaparib targeting DNA repair to acts synergistically in several non-small cell lung carcinoma cell lines [172]. This combinatorial therapy can be promising especially in patients with advanced breast and ovarian cancer-bearing BRCA1/2 (breast cancer 1/2) mutations [173].

#### 2.1.5. Summary

cisPt is used in vitro in concentration range approx. 0.5–300 μM. The levels in human plasma can reach up to 10 μg/mL (33 μM) which should be beared in mind when interpreting in vitro data. Continuous treatment, or longer incubation time, or high cisPt concentration of 20 mg/mL lead to complete inhibition of DNA synthesis [174]. The concentration range of 15–30 μM results in a block of DNA replication and transcription and triggers DNA damage response (DDR) signalization through ATM-CHK2, ATR-CHK1 DDR pathways resulting in p53-p21 driven cell cycle arrest or p53-mediated cell apoptosis [141,142,143,144]. However, in some cell lines also the synthesis of anti-apoptotic protein Bcl-2 was reported [143]. cisPt is in the majority of cell lines induces apoptosis above the concentration of approx. 2 μM [139,141,142,146]. cisPt block DNA replication [139,140,146] and inhibits RNA synthesis [140,175,176] and also influences the mitochondrial DNA synthesis and metabolism [147]. As a commonly used drug in clinics, many in vitro experiments have been conducted to address problems arising during treatment. Especially, the study of mechanisms underlying drug resistance [177], causes of toxic side effects [178], enhancement of synergistic effects [179] and ways how to improve drug delivery systems [180]. cisPt massively triggers the TLS repair pathways; defective FA proteins sensitise the cells towards this compound [181], defective MMR proteins establish cisPt resistance [103,182].

### 2.2. Aphidicolin

**Figure 3:**
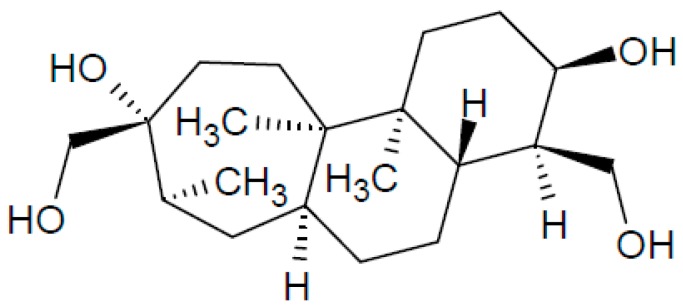
Aphidicolin structure.

Aphidicolin (APH) is a tetracyclin diterpenoid antibiotic isolated from *Nigrospora sphaerica* (Figure 3) which interferes with DNA replication by inhibiting DNA polymerases α, ε and δ [183]. Specifically, only cells in S phase are affected, whereas cells in other phases of the cell cycle are left to continue until the G1/S checkpoint, where they accumulate [184].

#### 2.2.1. Mechanism of DNA Damage Induction

APH binds to the active site of DNA polymerase α and rotates the template guanine, selectively blocking deoxycytidine triphosphate (dCTP) incorporation [185]. DNA polymerase α interacts with APH by its C18-binding OH group, APH forms a transient complex with polymerase and DNA [183]. The effect of APH on cell cultures is reversible if the cells are treated for no longer than 2 generations [186]. The exonuclease activity of APH-responding polymerases is only mildly affected, even at concentrations completely blocking the polymerase activity [183]. However, in the cell nucleus, the exonuclease activity is usually not retained because ternary complex APH–polymerase–DNA is formed and blocks the enzyme [183]; 3D structure of the complex can be found here [187].

Mechanistically, APH compromises the function of DNA polymerase, while helicase proceeds regularly (so called uncoupled/disconnected replicon), which leads to the generation of long stretches of single-stranded DNA [188]. The disconnected replicon is vulnerable structure prone for breakage preferentially at the so-called common fragile sites (CFSs) (also referred to as CFS expression) [189]. CFSs are specific genomic loci conserved in mammals generally prone to instability upon RS [190]. CFS expression is also common in precancerous and cancerous lesions [76]. Moreover, a causative role of CFS’s in cancer development has been suggested [191]. APH reproducibly causes damage at the same sites, and thus low doses of APH are used to define APH-inducible CFSs, of which there are over 80 described in the human genome [22,192]. Other CFS inducers (hydroxyurea, camptothecin, hypoxia and folate deficiency) are not so specific, nor efficient as APH [193,194]. Importantly, APH efficiently induces CFS expression only when the rate of polymerase is slowed down but not completely blocked. The optimum concentration range usually spans 0.1–1 μM [195] (and refer to Table 2). Apart from disconnected replicon, there might be other explanations for the extraordinary potency of APH to induce CFS-associated genomic instability. First, APH has been shown to increase the number of R-loops within certain CFSs, thus inducing replication/transcription collisions [196]. However, the mechanistic relationship between APH and increased R-loop formation is not clear. Second, re-licensing of replication origins is typical feature of oncogenic genetic backgrounds which are very prone to CFS expression. In such situations the CFS expression is explained as a result of DNA re-replication and subsequent collision of re-replicating forks within CFSs [10,197]. This phenomenon was studied in detail in yeasts at replication slow zones (analogs to CFSs in mammals) [198]. It is not clear whether the same re-licensing process is induced also by APH, however re-duplication would explain the reported APH-induced amplifications [191,199].

Prolonged treatment with low doses of APH induces cellular senescence response [74]. Interestingly, the most efficient doses were found to span the same range as doses used for CFS expression, which implies that CFSs might play a causative role in this process. Moreover, oncogene-induced senescence also displays increased CFSs-associated instability [10,197]. These phenotypical similarities between oncogenic stress and low doses of APH make this drug a good candidate for studying cellular processes in early stages of malignant transformation.

#### 2.2.2. Other Effects

APH is a very specific DNA polymerase inhibitor, APH does not interact with mitochondrial DNA polymerases [186] nor proteins [200], DNA, RNA, metabolic intermediates, nor nucleic acid precursor synthesis [184,200,201]. Contradictory results have been obtained regarding the effect of APH on DNA repair synthesis (DRS). According to a radiography method, APH does not influence DRS [200], although when DRS was induced by tumor necrosis factor (TNF) or UV irradiation, APH was observed to inhibit the process [202,203]. For references to particular studies using APH, refer to Table 2.

#### 2.2.3. Solubility

APH (MW 338.48 g/mol) is soluble in DMSO (up to 10 mg/mL), ethanol (up to 1 mg/mL) and methanol (freely), not soluble in water. The stability of the powder is 3 years at 2–8 °C, ethanol solution for a week at 2–8 °C, DMSO solution for 6 weeks at −20 °C [218].

#### 2.2.4. Medical Use

APH has limited use in clinical practice owing to its low solubility. Only APH-glycinate has so far been tested in clinical trial phase I. However, fast clearance from human plasma (no drug observed after 6–8 h of APH administration) and no anti-tumour activity was observed. Its use as a single agent or even in combination with other cytostatics is no longer being considered [219]. APH is metabolised by cytochrome P-450 dependent degradation [220]. APH and its derivatives are considered as potential therapeutics for parasitic diseases, e.g., Chagas disease [221].

#### 2.2.5. Summary

APH is used for in vitro studies in concentration range approx. 0.01 μM to 0.2 mM. APH is mainly used for cell-based experiments involving CFS expression [222], cell cycle synchronization [223], replication fork stability and restart studies [224] and for cellular senescence induction [74]. The threshold between replication fork stalling and slowing down is around 1 μM. Upon higher concentrations (5 μM–0.2 mM) APH was reported to stall the DNA polymerase, leading to S phase arrest. Upon lower concentrations, when the DNA polymerases are just slowed down, CFS expression can be observed. Usually, longer incubation times (approx. one population doubling) are used, so more cells within the population are affected. APH treatment causes a significant amount of DNA damage, leading to rapid ATR kinase activation. In the case of longer APH treatment also ATM is activated probably as a consequence of DSB formed within the stalled replication forks [207]. Prolonged APH incubation in the range of days up to weeks at low concentrations (0.2–1 μM) induces cellular senescence [74].

### 2.3. Hydroxyurea

**Figure 4:**
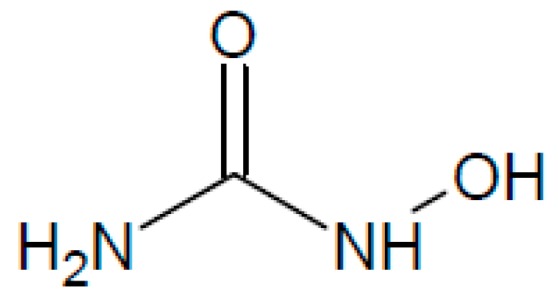
Hydroxyurea structure.

Hydroxyurea (HU) was first synthesised in the 19th century (Figure 4) and inhibits the incorporation of nucleotides by interfering with the enzyme ribonucleotide reductase (RNR) [225]. RNR converts nucleotide di- and tri-phosphates to deoxynucleotide di- and tri-phosphates, which is the rate-limiting step in nucleotide synthesis [226]. Without proper levels of dNTPs, DNA cannot be correctly replicated nor repaired [227].

#### 2.3.1. Mechanism of DNA Damage Induction

RNR is a large tetrameric enzyme comprising two R1 subunits and two small regulatory subunits R2 [228]. HU scavenges the tyrosyl radical of the R2 subunit which inactivates the RNR enzymatic activity [226]. Complete inhibition of RNR has been observed within 10 min after treatment with 0.1 mM HU and within 5 min after 3 mM of HU in murine 3T6 cells [229]. Consequently DNA synthesis is inhibited, selectively stopping the cells in S phase [230]. The inhibition is caused alterations in the dNTP pools. Each type of dNTP is affected in a different way. For example, after 280–560 μM HU treatment for 60 min, the dTTP pool was found to increase by 50%, whereas the dCTP pool is decreased by 50% [231]. HU slows down the initiation of replication and also the progression of replication forks. Moreover, after stopping the production of dNTPs, DNA repair and mitochondrial DNA synthesis are affected in all cells, regardless of the cell cycle stage [227]. HU treatment greatly affects the choice of replication origins and origin spacing in mammalian cells [232]. Although the mechanism of DNA damage induction may look similar to that for APH, HU induces a different spectrum of fragile sites, called early replicating fragile sites (ERFs) [233]. ERFs are also induced by c-Myc expression [11,12]. It was also reported that 10 μg/mL APH [234] (concentration that stalls the replication fork progression) leads to the generation of several kilobases long unwound DNA; however, HU treatment can generate only up to 100–200 nt long ssDNA [235].

#### 2.3.2. Other Effects

HU induces copy number variants (CNVs) with similar frequency and size distribution as APH [236]. It was reported for yeast cells, that HU alters Fe–S centres, enzyme cofactors catalysing oxidation-reduction reactions, which interferes with various metabolic enzymes and affects the redox balance of cells. Similar mechanism is proposed also for mammalian cells [237].

HU has been negatively tested for mutagenicity, measured by single nucleotide variation (SNV) and insertion/deletion frequency [238]. On the other hand, low doses of HU have been reported to induce DNA damage [239]. Therefore, it is possible that the compound possesses some pro-mutagenic potential (see also below). For references to particular studies using HU, refer to Table 3.

#### 2.3.3. Solubility

HU (MW 76.05 g/mol) is freely soluble in water at 100 mg/mL, soluble also in DMSO. The powder is stable at 4 °C for 12 months. Solutions are stable for 1 month at −20 °C (after defrosting, equilibration is recommended for 1 h at room temperature. It is recommended to prepare fresh solutions before use. HU decomposes in the presence of moisture; therefore, it is recommended that it is stored in air-tight containers in a dry atmosphere [259].

#### 2.3.4. Medical Use

HU is a commonly used medicine first approved by the FDA for the treatment of neoplastic disorders in the 1960s [260]. Common plasma levels of HU range 100–200 μM [261]. It is used for the treatment of sickle cell disease, essential thrombocytosis [262], myeloproliferative disorders and psoriasis [260] and is commonly indicated as a cytoreductive treatment in polycythemia vera [263] and others. Synergistic effects have been reported when it is used in combination with antiretroviral pills [264] and also in indicated cases with radiotherapy [265]. HU may be used as an anti-retroviral agent, especially in HIV (human immunodeficiency virus) patients. HU may cause myelofibrosis development with increased time of use and AML/MDS syndrome (acute myeloid leukaemia/myelodysplastic syndrome) [266]. Adverse side-effects have been observed, mainly myelosuppression [267]. A 17-year follow-up study of 299 patients treated with HU as a long-term therapy showed no difference in the incidence of complications such as stroke, renal disease, hepatic disease, malignancy or sepsis [268], suggesting that HU is well-tolerated. However, CNVs are generated at therapeutic doses of HU, and data from reproductive studies and studies on subsequent generations have so far been rather limited [236,268].

#### 2.3.5. Summary

HU is used in vitro approx. in the range 2 mΜ–5 mM. The most commonly used concentrations are around 2 mM. HU is used for cell cycle synchronization [269], replication fork stability studies [249,252], studies of recovery mechanisms after the release of RS [242] and checkpoint responses [241]. Lower concentrations are used for RS induction [254], induction of senescence [74], apoptosis [257], and repair pathways induction [217]. HU reaches plasma concentrations around 0.1 mM; this should be bear in mind when interpreting the data for clinical relevance [261]. The MRN (Mre11-Rad50-Nbs1) complex members Mre11 (Meiotic recombination 11) and Nbs1 (Nijmegean breakage syndrome 1) are required for efficient recovery of replication after treatment with replication stalling agents such as hydroxyurea [12]. HU causes rapid generation of ssDNA as indicated by RPA loading 40 min after treatment [270]. Subsequently, ATR-CHK1 signalling is activated, and HR repair pathway is induced.

Cells deficient in XRCC2 or other homologous recombination components exhibit hypersensitivity to HU [271]. It was reported that for hamster V79 cells, low concentrations of HU (5–10 μM) mimics the replication dynamics of untreated HR deficient cells [217]. Cellular senescence after long term replication stress caused by HU is dependent on p53-p21 signalling pathway and independent of p16 [74]. HU influences mutiple cellular pathways, e.g., JNK pathway, mitochondrial and peroxisome biogenesis, expression of several heat shock response proteins, autophagy pathways stimulation (beclin-1 expression), hemoglobin type F induction (in sickle cell disease, β-thalasemmia patients), etc. [272]. There are several cell lines that response to HU treatment in a specific manner, e.g., K562 cell line undergoes differentiation [253], T-cells activation is decreased [264], the morphology of vascular endothelial cells is affected [273].

### 2.4. Camptothecin

**Figure 5:**
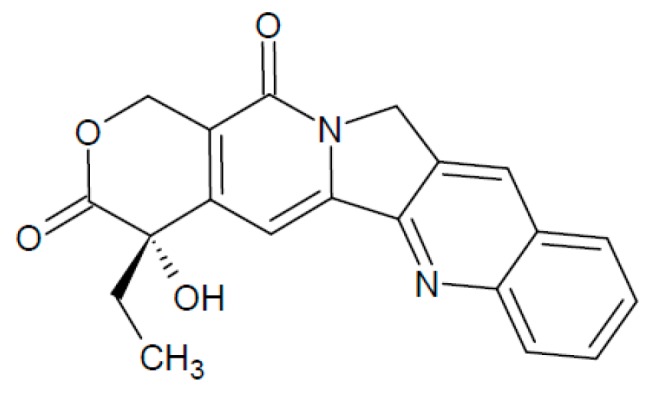
Camptothecin structure.

Camptothecin (CPT) is a pentacyclic quinoline alkaloid first isolated from the Chinese tree *Camptotheca acuminata* (Nyssaceae) by Wall et al. [274] (Figure 5). CPT has a unique intracellular target, topoisomerase I (TopoI), a nuclear enzyme that reduces the torsional stress of supercoiled DNA [24]. This activity enables specific regions of DNA to become sufficiently exposed and relaxed to facilitate essential cellular processes, such as DNA replication, recombination and transcription [275].

#### 2.4.1. Mechanism of DNA damage induction

TopoI binds covalently to double-stranded DNA through a reversible transesterification reaction, generating a SSB [276], 3D structure can be found here [277]. This so-called TopoI–DNA cleavage complex (Top1cc) facilitates the relaxation of torsional strain in supercoiled DNA, either by allowing passage of the intact single strand through the nick or by free rotation of the DNA around the uncleaved strand [278]. CPT covalently and reversibly stabilises the normally transient DNA Top1cc by inhibiting religation of the scissile strand, thereby prolonging the half-life of Top1cc and increasing the number of DNA SSBs [279,280]. Moreover, trapping of the enzyme on the DNA leads to rapid depletion of the TopoI pool [281]. The effect of CPT is readily reversible after removal of the drug. However, prolonged stabilisation of Top1cc can cause multiple problems. Firstly, failure to relieve supercoiling generated by such processes as transcription and replication can lead to RS by creating torsional strain within the DNA [279,281,282]. Furthermore, the collision of the RF with the ternary drug-enzyme-DNA complex generates DSBs with serious cellular consequences, including cell death [283,284].

Because ongoing DNA synthesis is important for CPT-induced cytotoxicity, CPT is considered an S phase-specific drug. The repair of CPT-induced DSBs involves multiple DNA damage repair proteins. Recent studies have highlighted that functional cooperation between BRCA2, FANCD2, RAD18 and RAD51 proteins are essential for repair of replication-associated DSBs through HR. Loss of any of these proteins causes disruption of HR repair, chromosomal aberrations and sensitization of cells to CPT [285]. A close link between CPT and HR has also been demonstrated in experiments measuring sister chromatid exchange events (SCEs), which are common consequence of elevated HR repair process and found to be induced by low doses of CPT [270]. CPT is applied in early S phase cells for triggering G2 arrest accompanied by blockage of the p34cdc2/cyclin B complex, a consequence of either DNA breakage, the arrest of the replication fork or both [286]. In addition, CPT driven TopoI–DNA cleavable complex and associated strand breaks were shown to increase transcription of the c-Jun early response gene, which occurs in association with internucleosomal DNA fragmentation, a characteristic mark of apoptosis [287]. Noncytotoxic concentrations of CPT can induce the differentiation of human leukaemia cells [288], and an antiangiogenic effect is suggested [289,290]. Interestingly, when used in combined treatment with APH, CPT reduces the APH-induced RPA (an indicator of ssDNA) signal and has a rescuing effect on CFS expression [291]. For references to particular studies using CPT, refer to Table 4.

#### 2.4.2. Solubility

CPT (MW 348.35 g/mol) is soluble in DMSO (up to 10 mg/mL), methanol (40 mg/mL), 0.1 N sodium hydroxide (50 mg/mL) or acetic acid, insoluble in water. At higher concentrations, heating is required to dissolve the product completely (approx. 10 min at 95 °C), but some precipitation occurs upon cooling to room temperature [301].

#### 2.4.3. Medical Use

CPT cannot be used in clinical practice because of its poor solubility in aqueous solutions, instability and toxicity, but modifications at selected sites have improved the pharmacologic and activity profile [283]. Currently, three water-soluble CPT-derivates, i.e., irinotecan (CPT-11), topotecan (TPT) and belotecan (CKD-602), are available for cancer therapy. However, despite their selectivity for TopoI and unique mechanism of action, they all have critical limitations. In particular, they become inactivated against TopoI within minutes at physiological pH due to spontaneous lactone E-ring opening [302] and diffuse rapidly from the TopoI–DNA cleavage complex due to their noncovalent binding. To overcome these problems, five-membered E-ring CPT-keto non-lactone analogues S38809 and S39625 have been synthesised and selected for advanced preclinical development based on their promising activity in tumour models. Their chemical stability and ability to produce high levels of persistent Top1cc makes them useful candidates for future treatment [303].

#### 2.4.4. Summary

Camptothecin is used in concentration range 2.5 nM up to 20 μM. CPT is a potent DSBs inducer in a wide concentration range, approx. 10 nM–10 μM. Upon higher concentration (20 μM–10 μM), CPT was reported to be cytotoxic, increasing cell apoptosis via DNA fragmentation predominantly in S phase cells with ongoing DNA synthesis [292,293]. The most frequently used concentration of 1 μM CPT was shown to block DNA synthesis and induce DSBs resulting from the collision of RF due to prolonged stabilisation of TopoI DNA cleavage complex. The main implication of lower CPT concentrations is the induction of replication fork slowing and reversal, as a rapid response to TopoI inhibition is the increase in topological stress of DNA locally [300]. CPT activates predominantly ATR-CHK1 and ATM-CHK2 signalling, and leading to G2 checkpoint arrest [300]. Even at low doses of CPT HR repair pathway is triggered.

### 2.5. Etoposide

**Figure 6:**
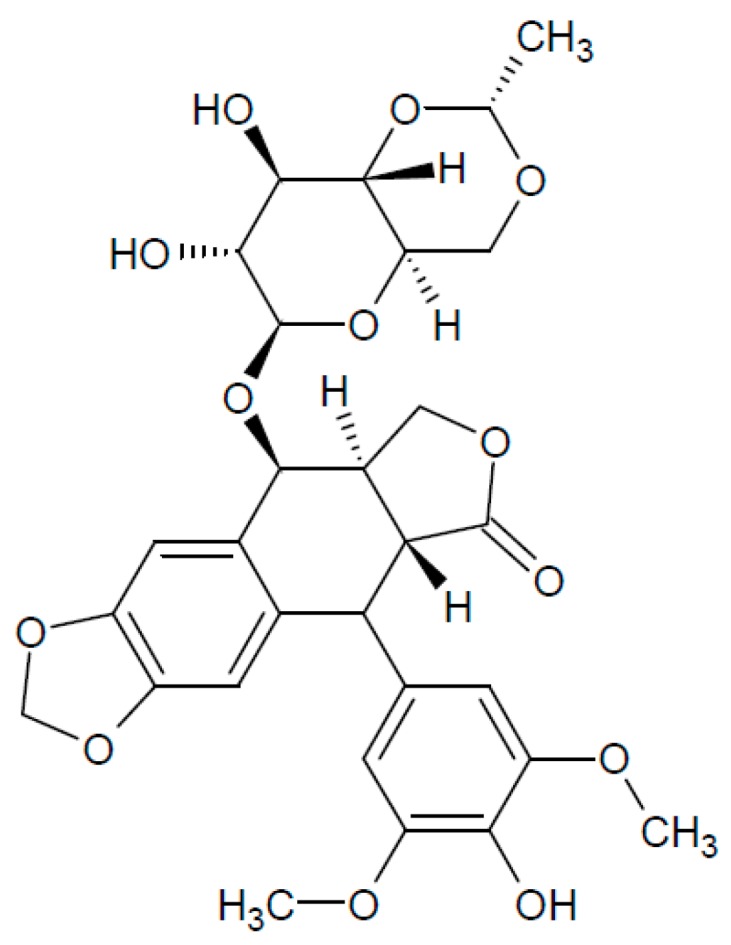
Etoposide structure.

Etoposide (ETP) is a derivative of podophyllotoxin first synthetised in 1966 and approved for treatment as an antineoplastic agent in 1983 [304]. ETP structure comprises of polycyclic A–D rings, an E-ring and aglycone core (Figure 6). ETP compromises the proper function of the enzyme topoisomerase II (TopoII), 3D structure can be found here [305]. TopoII performs cleavage of both strands of a DNA duplex and enables passage of a second intact duplex through the transient break, ATP is used to power the strand transition [306]. As a result, relaxation, unknotting and decatenation of DNA are achieved enabling processes like replication and transcription [25].

#### 2.5.1. Mechanism of DNA Damage Induction

Two modes of action were suggested for ETP to interfere with TopoII [25]. As a poison, it stabilises TopoII:DNA complexes, whereas as an inhibitor ETP interacts with the catalytic site of TopoII, decreasing the number of active cleavage complexes [307]. ETP acts as a poison by stabilizing the cleavage complex of TopoII via decoupling the key catalytic residues, thus preventing the religation of cleaved DNA ends [308]. As a result, the number of TopoII-associated DNA breaks are increased [309]. ETP’s A, B and D-rings mediate the drug-enzyme interaction, whereas the aglycon core binds to DNA [262,308]. E–ring substituents are important for ETP activity but do not contribute to ETP-enzyme binding [310]. ETP is metabolised by cytochrome P3A4 (CYP3A4) to two metabolites, ETP-quinone and ETP-catechol. Both active against the TopoII enzyme. ETP-quinone is approx. 100× more efficient at inhibiting TopoII than ETP. ETP-quinone can block binding of the enzyme to DNA by stabilisation of the N-terminal clamp [307]. In cases where the enzyme still binds to DNA, the metabolite can stabilise the enzyme:DNA complex by inhibiting the religation step thus leading to higher levels of DSBs [307]. The ETP-catechol metabolite works similarly to the parent compound but can also be oxidised to the quinone [311]. ETP induces DSBs directly in all phases of the cell cycle, as observed by γH2AX foci formation (a marker of DSBs) [312,313]. ETP does not require S-phase to induce damage, but ongoing replication enhances its cytotoxic effect [314]. ETP causes disassembly of replication factories (sites of ongoing replication), as measured by the distribution of proliferating cell nucelar antigen protein (PCNA) [315]. Moreover, the cytotoxic effect of ETP is partially reduced by inhibitors of DNA synthesis, such as APH and HU [316]. There are two isoforms of the TopoII enzyme in mammals, called TopoIIα and TopoIIβ, sharing 68% homology [317]. TopoIIα activity is upregulated during cell cycle progression, peaks in mitosis and is essential for proliferating cells [318]. TopoIIβ is needed during transcription and DNA repair, and its levels are more stable during the cell cycle [319]. ETP is not selective between these two paralogs, and the inhibition of TopoIIβ is believed to be the reason for ETP therapy-related secondary malignancies [320]. TopoIIα seems to be a better target for therapy. Therefore, new compounds and analogues of ETP have been synthesised to be selective only for TopoIIα [321].

#### 2.5.2. Other Effects

A strong mutagenic effect has been measured for ETP in mammalian cells by several assays, e.g., HPRT assay (hypoxanthine phosphoribosyl transferase), SCE and detection of mutations at the locus of the adenosine kinase gene [322]. In prokaryotic organisms (*E. coli*, *Salmonella typhimurium*), no significant genotoxic effect was observed [322]. For references to particular studies using ETP, refer to Table 5.

#### 2.5.3. Solubility

ETP (MW 588.56 g/mol) is soluble in organic solvents (ethanol, methanol, DMSO), poorly soluble in water. It is recommended that stock solutions in organic solvents be diluted so 0.1% organic solvent is present in the final solution. The stability in aqueous solution is best at pH 4–5, but it can be improved by adding polysorbate 80 (Tween80), polyethylene glycol 300, citric acid and alcohol. ETP is unstable under oxidative conditions [338]. Under acidic conditions (pH < 4), the glycosidic linkage and lactone ring are hydrolysed, whereas, under basic conditions (pH > 6), *cis*-lactone epimers are formed [304]. Aqueous solutions are stable for several hours, depending on the concentration of the solution but irrespective of the temperature. ETP is sensitive to UV irradiation, both in solution and as a powder [338].

#### 2.5.4. Medical Use

According to pharmacokinetic studies, plasma levels of ETP peak at concentrations of 20–70 μM [339]. ETP is approved for the treatment of refractory testicular tumors and small cell lung cancer. Various chemical modifications with potential higher efficacy have also been tested for clinical use, e.g., 4′-phosphorylation or 4′-propyl carboxy derivatives [340]. In the field of so-called personalised medicine, combined subsequent treatment of ETP and cisPt has been shown to be beneficial for patients suffering from ERCC1-incompetent lung adenocarcinoma [341]. ETP is reported to cause therapy-related leukaemias [320] and specific chromosomal translocations. Chromosomal rearrangements at the 11q23 chromosome band were found in patients and seemed to be related to the CYP3A4 metabolic conversion of ETP [342]. In mouse embryonic stem cells, an increase in fusion chimeric products was observed at a 1.5 kb “hot spot” between exons 9 and 11 (analogous region to MLL (mixed lineage leukaemia) breakpoint cluster in human leukaemia) [343]. MLL gene encodes lysine (K)-specific histone methyltransferase 2A therefore influencing histone methylation and gene expression [344]. Leukaemogenic MLL translocations lead to expression of MLL fusion proteins. Patients with such translocations exhibit poor prognosis [345]. MLL fusion proteins are efficient in transforming the hematopoetic cells into leukaemia stem cells [346]. Many studies have attempted to solve the adverse side effects of ETP treatment and understand the underlying molecular mechanisms, e.g., multi-drug resistance [347], or unwanted toxicity [348]. The search for compounds that may improve ETP treatment usually starts with cell-based experiments, e.g., protective compounds shielding healthy cells [349], compounds selectively enhancing ETP toxicity [350] or targeted delivery [351].

#### 2.5.5. Summary

ETP is commonly used for the induction of apoptosis [352]. Indeed, several studies reported that higher doses of the compound (25–100 µM) activate apoptosis, mostly in a manner dependent on p53 [325,326,327,329]. Prolonged treatment at lower concentrations of ETP can also lead to induction of the p53 pathway, cell cycle arrest, senescence and apoptosis [145,325,330,335,337]. ETP induces the formation of irreversible DNA–TopoII cleavage complexes (TopoIIcc) and DNA damage regardless of concentration or incubation time [323,324,329,330,331,332,334,353]. The initial displacement of TopoIIcc requires the coordinated action of several processes, such as cleavage by the 5’-tyrosyl DNA phosphodiesterase (TTRAP) and proteasome-dependent degradation of TopoII [354,355]. Furthermore, the MRN complex, CtIP (RBBP8 protein) and BRCA1 play a critical role in the removal of such DNA-protein adducts [356]. The remaining DNA lesions are often referred as DSBs, which are accompanied by the activation of ATM-mediated signalling or repair pathways, usually quantified by the formation of γH2AX [323,324,329,330,331,332]. However, several studies argue against the ability of ETP to primarily induce DSBs, showing that majority of the DNA lesions formed upon ETP treatment are SSBs [323,329]. Despite the discrepancy, pathways engaged in DSB repair are activated after the exposure to the drug, and among them, NHEJ is seemingly predominant [329,356,357,358]. ETP used in relatively high concentration (20–25 µM) might lead to persistent or irreparable DSB formation [329,331,332].

## 3. Conclusions

Replication stress is a significant contributor to genomic instability, a major factor for the conservation of mutations [1], relevant promoter of tumourigenesis [8] and also one of the main features of cancer cells [76]. Owing to its complexity, replication can be disturbed by multiple mechanisms. In this review, we focused on several compounds known to be RS inducers and often used in cell-based assays. Some of the compounds have been shown to be effective in cancer treatment. Importantly, the chemicals have been primarily chosen to cover various mechanisms of action, resulting in different treatment-induced phenotypes resembling those of RS in carcinogenesis. Induction of RS in vitro, e.g., by chemicals inducing DNA damage, is a crucial research tool. Precise knowledge about the mechanism of DNA damage induction and cellular pathways involved in the RS response is particularly important for the development of appropriate cellular assays for investigating carcinogenesis and cancer treatment. The above-mentioned publications in separate compound-related tables were chosen to help with the practical aspects of such assay design. Dose and time-dependent effects related to the genetic backgrounds (i.e., dependent on the cell line used) and proper readout are important issues for experiment design. Moreover, other practical information has been included so that readers can use this review as a brief guide for choosing an appropriate model and dose scheme for cell-based studies.

## Figures and Tables

**Table 1:** Effects of various cisplatin treatments in vitro.

Concentration	Incubation Time	Observed Effect	Cell Line	Reference
300 μM	2 h	increase in polyADP ribosylation	O-342 rat ovarian tumour cells	[137]
100 μM	2 h before IR	sensitization to γ-radiation	hypoxic V-79 Chinese hamster cells	[138]
100 μM	2 h	increase in polyADP ribosylation	CV-l monkey cells	[139]
<20 μg/mL (<66 μM)	5 h	block of rRNA synthesis block of DNA replication	Hela	[140]
15 μM	1 h	induction of SCE (sister chromatid exchange) decreased cell survival	6 primary human tumour cell culture	[141]
10–30 μM	24 h, 48 h	induction of apoptosis	224 (melanoma cells) HCT116	[142]
10 μM	24 h	increase in antiapoptotic Bcl-2 mRNA synthesis (regulated by PKC and Akt2)	KLE HEC-1-A Ishikawa MCF-7	[143]
2–10 μM	72 h	induction of apoptosis	224 (melanoma cells) HCT116	[142]
5 μM	24 h	increase in p53 stability activation of ATR increased p53(ser15) phosphorylation	A2780	[144]
5 μM	24 h	activation of p21 activation of CHK2 increased p53(ser20) phosphorylation	HCT116	[144]
5 μM	24 h	induction of mitochondrial reactive oxygen species (ROS) response	A549 PC3 MEF	[143]
2 μM	24 h	G2/M arrest, subapoptic damage	MSC	[145]
>2 μM	24 h	decreased proliferation rate induction of apoptosis	TGCT H12.1 TGCT 2102EP	[145]
1–4 μg/mL	2 h	block of DNA synthesis	L1210/0 cells	[146]
		block of transcription		
		G2 arrest		
		apoptosis		
2 μg/mL	48 h 144 h, 168 h	inhibition of mtDNA replication inhibition of mitochondrial genes transcription	Dorsal root ganglion (DRG) sensory neurons	[147]
1 μg/mL	2 h	transient G2 arrest	Hela	[148]
3.0 μM	4 h before	block of NHEJ	A2780	[138]
0.2–0.8 μM	IR 0.5 Gy	cisPt-IR synergistic interaction	MO59J MO59K	[138]
	4 h			
1–2.5 μM	24 h–48 h	block of DNA replication followed by cell apoptosis	Hela	[149]
0.3–1 μM	overnight	inhibition of RNA polymerase II-dependent transcription	Hela XPF	[144]
0.6 μM	2 h	90% reduction in clonogenic capacity detected after 7 days CHK1 phosphorylation causing CHK1 dependent S phase arrest	Hela	[148]
0.5 μM	24 h 48 h	loss of telomeres (TEL), or TEL repeats cell death	Hela	[139]

ATR: Ataxia telangiectasia Rad3-related; Bcl: B-cell lymphoma; CHK1: checkpoint kinase 1; CHK2: checkpoint kinase 2; IR: ionizing radiation; mtDNA: mitochondrial DNA; NHEJ: non-homologous end-joining; PKC: protein kinase C; polyADP: poly adenosine diphosphate; rRNA: ribosomal RNA.

**Table 2:** Effects of various aphidicolin treatments in vitro.

Concentration	Incubation Time	Observed Effect	Cell Line	Reference
0.2 mM	16 h, 10 h	formation of anaphase bridges and micronuclei	HeLa	[204]
30 μM	6 h	stalled replication forks	HCT116	[205]
30 μM	6 h	stalled replication forks	PD20 cells Bloom syndrome cells	[206]
5 μg/mL (14.3 μM)	4 h	DNA repair synthesis inhibition sensitization towards TNF treatment	L929 ovarian cancer cells A2780	[202]
5 μg/mL (14.3 μM)	2–8 h	S phase arrest kinetics and mechanism study	RKO 293T MEF	[207]
2.5 μg/mL (7.15 μM)	1 h	inhibition of DNA synthesis and DNA repair	Normal and XPA deficient human fibroblasts	[203]
10 μM	15 h	cell cycle synchronisation at the G1/S boundary	REF-52 HeLa	[208]
5–25 μM	24 h	inhibition of replicative polymerases	Werner syndrome cells Bloom syndrome cells	[209,210]
1 μM	1–24 h	CFS induction	HEK293T	[210]
1 μM	24 h	CFS induction	MEF HeLa	[211]
0.5 μM	2 h	transient attenuation of DNA synthesis,	DT40	[212]
0.1 μM	24 h	study of chromosome integrity and replication		
0.4 μM	24 h	CFS induction	U-2 OS	[213]
0.1 μM 0.2 μM	16 h	replication stress observed on telomeres	hESC (UCSF4)	[214]
0.2 μM	2 weeks	irreversible senescence induction	REF-52	[74]
0.2 μM	24 h	CFS induction	BJ-hTERT	[215]
0.05 μM 0.4 μM	24 h	CFS induction	Werner syndrome fibroblasts AG11395 cells	[216]
0.3 μM	48 h	increased incidence of mitotic extra chromosomes replication stress	V79 hamster cell lines	[217]
0.3 μM	72 h	replication stress	Human fibroblasts HGMDFN090	[199]
2 μg/mL	not indicated	replication block	BJ BJ-tert HMEC	[197]
0.2 μM	7–24 h	cell synchronization	HeLa	[184]

CFS: common fragile site; TNF: tumour necrosis factor.

**Table 3:** Effects of various hydroxyurea treatments in vitro.

Concentration	Incubation Time	Effect	Cell Line	Reference
200 mM	2 h	replication block	yeast cells	[240]
10–200 mM	3 h	replication block replication fork (RF) restart	yeast cells	[241]
5 mM	1 h	replication block	HEK293	[242]
2 mM	3 h	replication block		
50 μM–5 mM	40 min–2 h	replication stress	293T mouse ES cells	[243]
2 mM	1 h, 24 h	replication stress replication block	HCC1937 MCF7	[244]
2 mM	16 h	replication block	HEK293	[245]
2 mM	24 h	DNA damage induction during S phase	U-2 OS 293T	[246]
2 mM	15 h	replication block cell cycle synchronisation at the G1/S boundary	REF-52 HeLa	[208]
2 mM	5 h	dNTP depletion	REF52	[74]
2 mM	3 h	chromosomal aberrations FANCD2 pathway involvement	lymphoblastoid cell lines	[247]
1 mM	overnight	replication block	MCF7	[248]
0.5 mM	5 h–10 h	replication block	U-2OS	[249]
2 mM	2 h–24 h	replication block		
0.5 mM	90 min	nucleotides depletion stalled RF w/o DSBs formation	MEF	[250]
0.1–0.5 mM	2 h–72 h	γ-globin gene expression	K562	[251]
0.1–0.5 mM	2 h–8 h	replication stress	PC3	[252]
0.2–0.4 mM	4 days	cell differentiation ERK signalling pathway inhibition p38 signal transduction activation	K562	[253]
0.3 mM	10 days	microsatellite instability upon FANCJ depletion	GM08402 HeLa PD20F	[254]
0.15–0.2 mM	2 weeks	irreversible senescence induction	REF-52	[74]
0.2 mM	2 h–7 h	replication stress	MEF	[255]
0.15 mM	2 h	p53 activation	REF52	[74]
50–200 μM	20 h	HIF1 induction eNOS induction	HUVEC	[256]
25–200 μM	72 h	induction of apoptosis	AML cell lines (MV4-11, OCI-AML3, MOLM-13, and HL-60)	[257]
5 μM–0.5 mM	48 h	replication stress	V79 hamster cells	[217]
2 μM	12 h	replication stress	H1299	[258]

dNTP: deoxynucleotide triphosphate; DSBs: double-strand breaks; eNOS: endothelial nitric oxide synthase; ERK: extracellular signal-regulated kinases; FANCD2: Fanconi anaemia complementation group D2; FANCJ: Fanconi anaemia complementation group J; HIF: hypoxia induced factor 1.

**Table 4:** Effects of various camptothecin treatments in vitro.

Concentration	Incubation Time	Observed Effect	Cell Line	Reference
20 μM	30 min	DNA fragmentation in G1 and S phase cells	Hela	[292]
10 μM	24 h	increase in cell sensitivity to TRAIL-mediated apoptosis	Hep3B	[293]
10 μM	4 h	formation of replication mediated DNA DSBs	HT29	[294]
5 μM	60 min	inhibition of RNA synthesis	CSA	[295]
1 μM	60 min	inhibition of DNA synthesis	CSB	[296]
1 μM	60 min	replication block DSB formation cell death	U2OS	[297]
1 μM	60 min	formation of stabilised TopoI-cc complex DSB formation phosphorylation of CHK1 (S317) CHK2 (T68), RPA (S4/S8)	HCT116	[294]
1 μM	60 min	inhibition of DNA replication suggested DNA DSB formation	L1210 mouse lymphoblastic leukaemic cells	[293]
200 nM–1 μM	50 min	DSB formation	CSB	[298]
100 nM–10 nM	60 min	DSB formation	HCT116	[299]
25 nM	60 min	checkpoint activation (ATM-CHK2, ATR-CHK1) replication fork stalling replication fork reversal formation of specific DNA structures	U-2O-S	[300]
10 nM–100 nM	60 min	inhibition of EIAV (equine infectious anemia virus) replication	CF2Th	[295]
10 nM–20 nM	60 min	inhibition of HIV-1 replication block of viral protein expression	H9	[281]
6 nM	6 h	accumulation of cells in early S phase	Normal lymphocytes	[296]
	24 h	apoptosis, DNA fragmentation	MOLT-4	
6.25 nM	48 h	specific suppression of oral cancer cells growth	KB oral cancer cells	[281]
2.5 nM	48 h	increase in SCE upon depletion of Fbh1 helicase	BJ	[281]

ATM: Ataxia telangiectasia mutated; HIV: Human immunodeficiency virus; RPA: replication protein A; SCE: sister chromatid exchange; TopoI-cc: Topoisomerase I cleavage complex; TRAIL: TNF alpha related apoptosis inducing ligand, TNF: tumour necrosis factor.

**Table 5:** Effects of various etoposide treatments in vitro.

Concentration	Incubation Time	Effect	Cell Line	References
up to 450 µM	40 min	SSB and DSB formation, induction of H2AX phosphorylation with slow kinetics	SV-40 transformed human fibroblasts G361	[323]
1–100 µM	30 min	formation of TopoII-blocked DSBs, activation of ATM-mediated repair	MEF HEK293T BJ1 AT	[324]
2–100 μM	6 h–48 h	senescence, apoptosis induction of p53 response	HepG2 U2OS	[325]
2–100 μM	1–3 h	disassembly of replication factories	AT1 BR AT3 BR HeLa	[315]
50–100 μM	3–6 h/16 h	apoptosis (activation of intrinsic (mitochondrial) pathway)	Hela HCT116	[326]
50 μM	15 h	apoptosis	BJAB Hut78	[327]
50 μM	48 h	growth arrest (accumulation of cells at G2/M boundary) induction of p53 response	MCF-7 ZR75-1 T-47D	[328]
25 µM	1 h	SSB and DSB formation γH2AX, pATM, pDNA-PKcs, MDC1 foci formation persisting DSBs cell death	HeLa HCT116	[329]
20 μM	16 h	increase in γH2AX levels reduction of proliferation rate (accumulation of cells in S and G2/M boundary)	U2OS	[330]
20 μM	1 h	repairable DSBs	HEK293T COS-7 BJ-hTERT H1299	[331,332]
	16 h	irrepairable DSBs, ATM-dependent HIC1 SUMOylation, induction of p53-dependent apoptotic response		
20 µM	1–5 h	apoptosis	A549 HeLa, T24	[333]
10 μM	1 h	DNA damage induction	A549	[334]
1–10 μM	48 h	apoptosis	HCC1937 BT-549	[335]
8 μM	1 h	induction of p53 response,	SH-SY-5Y SH-EP1	[336]
0.75–3 µM	72 h	senescence, apoptosis	A549	[337]
0.75 μM	24 h	cell cycle arrest in G2/M phase, DNA damage induction, induction of p53 response	MSC TGCT H12.1 TGCT 2102EP	[145]

DSBs: Double strand breaks; HIC1: Hypermethylated In Cancer 1; MDC1: Mediator of DNA Checkpoint 1; pATM: phosphorylated Ataxia elangiectasia Mutated; pDNA-PKcs: phosphorylated DNA Protein Kinase catalytic subunit; SSB: single-strand DNA break; TopoII: Topoisomerase II.
